# Severe Combined Dyslipidemia With a Complex Genetic Basis

**DOI:** 10.1177/2324709619877050

**Published:** 2019-09-20

**Authors:** Ryan Le, Minan Abbas, Adam D. McIntyre, Robert A. Hegele

**Affiliations:** 1 Western University, London, Ontario, Canada

**Keywords:** dyslipoproteinemia, xanthoma, apolipoprotein E, DNA mutations, DNA sequencing

## Abstract

*Background.* Familial dysbetalipoproteinemia (also known as type 3 hyperlipoproteinemia) is typically associated with homozygosity for the apolipoprotein E2 isoform, but also sometimes with dominant rare missense variants in the *APOE* gene. Patients present with roughly equimolar elevations of cholesterol and triglyceride (TG) due to pathologic accumulation of remnant lipoprotein particles. Clinical features include tuberoeruptive xanthomas, palmar xanthomas, and premature vascular disease. *Case.* A 48-year-old male presented with severe combined dyslipidemia: total cholesterol and TG were 11.5 and 21.4 mmol/L, respectively. He had dyslipidemia since his early 20s, with tuberous xanthomas on his elbows and knees. His body mass index was 42 kg/m^2^. He also had treated hypertension, mild renal impairment, and a history of gout. He had no history of cardiovascular disease, peripheral arterial disease, or pancreatitis. Multiple medications had been advised including rosuvastatin, ezetimibe, fenofibrate, and alirocumab, but his lipid levels were never adequately controlled. *Genetic Analysis.* Targeted next-generation sequencing identified (1) the *APOE* E2/E2 homozygous genotype classically described with familial dysbetalipoproteinemia; (2) in addition, one *APOE* E2 allele contained the rare heterozygous missense variant p.G145D, previously termed apo E-Bethesda; (3) a rare heterozygous *APOC2* nonsense variant p.Q92X; and (4) a high polygenic risk score for TG levels (16 out of 28 TG-raising alleles) at the 82nd percentile for age and sex. *Conclusion.* The multiple genetic “hits” on top of the classical *APOE* E2/E2 genotype likely explain the more severe dyslipidemia and refractory clinical phenotype.

Familial dysbetalipoproteinemia (FDBL) or type 3 hyperlipoproteinemia (HLP) is a rare disorder that is usually associated with homozygosity for *APOE* E2/E2.^1-5^ Apolipoprotein (apo) E is a component of chylomicrons and their remnants, very low-density lipoprotein (VLDL), and intermediate density lipoprotein (IDL).^1-5^ Apo E mediates clearance of these lipoproteins and also plays an independent role in lipid metabolism in the brain.^6^ Three common isoforms of apo E, namely, E4, E3, and E2, differ by single amino acid changes encoded by common polymorphisms within the *APOE* gene.^5^ E2 has cysteine at amino acid residues 130 and 176 (formerly 112 and 158), in contrast to common E3, which has cysteine and arginine at residues 130 and 176, respectively.^5^ The cysteine at residue 176 in E2 leads to reduced binding affinity for cell surface receptors compared with E3.^2-5^ E2 allele frequency is ~10%, so that ~1% of people are homozygous for E2/E2. Because only ~10% of E2/E2 homozygotes develop FDBL,^2-5^ a second “hit” such as obesity, hypothyroidism, renal disease, estrogen deficiency, diabetes, or another genetic mutation is required for clinical expression.^2-5^ Occasionally, extremely rare missense mutations or deletion-duplication variants that impact on other amino acid residues of apo E can lead to compromised protein function, presenting clinically as FDBL.^2-5^

Clinical features of FDBL include tuberous and palmar xanthoma, plus increased risk of premature coronary and peripheral artery disease.^2-5^ Patients classically show equimolar elevations in serum total cholesterol (TC) and triglyceride (TG),^2-5^ due to massively elevated IDL. IDL is a normally a minor lipoprotein fraction but it accumulates in FDBL patients due to compromised particle binding from E2/E2 homozygosity. IDL is a remnant lipoprotein with atherogenic potential.^7^ FDBL patients often respond well to treatment with lifestyle modification and drugs such as fibrates, niacin, statins, and fish oil.^2-5^ In this article, we report a patient with severe refractory combined dyslipidemia and clinical features pathognomonic for FDBL, but with a complex underlying genetic architecture.

## Subject and Methods

**Figure 1.**
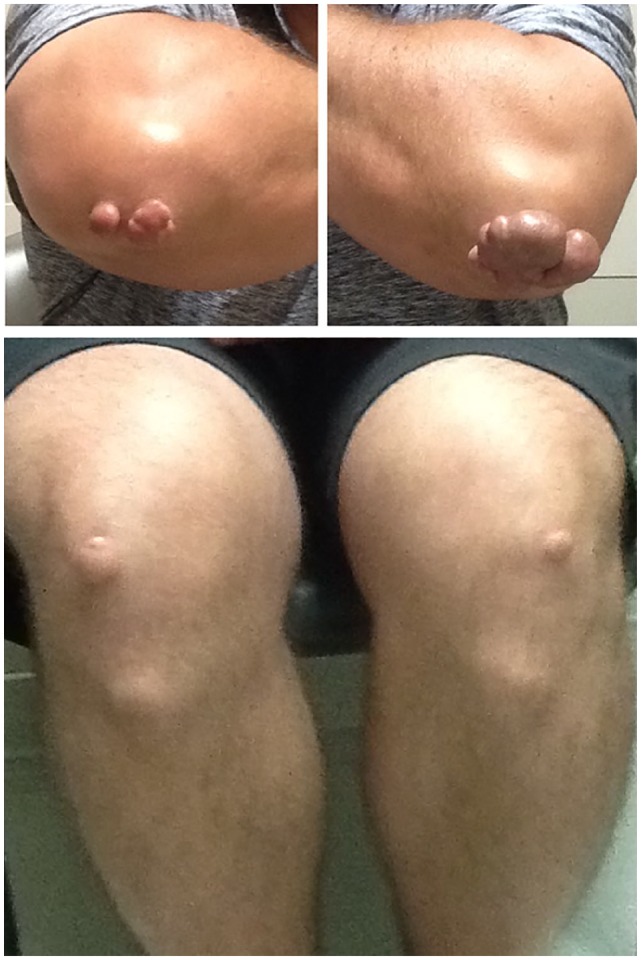
Tuberoeruptive xanthomas observed on the knees and elbows of the patient described in text.

A 48-year-old male of European ancestry was referred to Lipid Genetics Clinic with refractory combined dyslipidemia. In his early 20s, he presented with combined hyperlipidemia and tuberous xanthomas on his elbows. Treatment with fenofibrate and various statins resulted in only mild improvement of his lipid profile. He discontinued all medical treatment between 2010 and 2016, but returned due to worsening of tuberous xanthomas on his elbows and new bilateral knee xanthomas (Figure 1). Although weight loss and adherence to a low-fat diet were constantly reinforced, the patient failed to comply with this advice. Fenofibrate, atorvastatin, and ezetimibe were prescribed, but compliance was inconsistent due to leg cramps. Nicotinic acid was never attempted to our knowledge. Alirocumab 150 mg subcutaneously every 2 weeks was given between September 2017 and March 2018, with minimal effect on his lipid profile. He had no history of myocardial infarction, stroke, transient ischemic attack, pancreatitis, or symptoms of peripheral arterial disease such as intermittent claudication. He had no history of secondary factors such as multiple myeloma, systemic lupus erythematosis, paraproteinemia, or use of corticosteroids or other hormones. He was a nondrinker.

His medical history included treated hypertension, mild renal impairment with moderate proteinuria, and a remote history of gout. There was no family history of dyslipidemia. He was taking olmesartan 40 mg daily, hydrochlorothiazide 25 mg daily, and allopurinol 300 mg daily, and had recently stopped taking alirocumab. He smoked currently, with a 15 pack-year smoking history. On examination, his weight, height, blood pressure, and heart rate were 119.1 kg, 168 cm, 136/96 mm Hg, and 96 beats per minute, respectively. He had tuberous xanthomas on his elbows and knees bilaterally (Figure 1). There were no other xanthomas, nor were there xanthelasmas, arcus cornealis, or lipemia retinalis. Examination of his cardiovascular, respiratory, neurological, and abdominal systems was unremarkable.

Laboratory investigations in June 2010 obtained from medical records showed markedly elevated TC and TG at 16.2 and 16.8 mmol/L, respectively; high-density lipoprotein (HDL) cholesterol was 0.63 mmol/L, and non-HDL cholesterol was 15.6 mmol/L. Screening for secondary factors showed a hemoglobin A_1C_ of 5.4%, thyroid stimulating hormone of 2.83 mIU/L (normal range = 1-4 mIU/L), alanine transaminase of 43 U/L (normal <46 U/L), and a creatinine of 116 µmol/L (normal <110 µmol/L), with a urinary albumin to creatinine ratio of 142 mg/mmol (normal <30 mg/mmol/L).

In December 2017, TC and TG on rosuvastatin 20 mg and fenofibrate 145 mg daily were 13.6 and 19.9 mmol/L, respectively, with HDL and non-HDL cholesterol of 0.71 and 12.8 mmol/L, respectively. With addition of alirocumab, TC and TG were 11.1 and 29.0 mmol/L, respectively, with HDL and non-HDL cholesterol of 0.62 and 10.5 mmol/L, respectively. Plasma apo A-I was 1.3 g/L (normal range = 1.2-1.4 g/L), and apo B was 0.50 g/L (target <0.8 g/L). Direct LDL cholesterol measurement was never attempted. Carotid ultrasound in 2018 showed intima media thickness at the 50th percentile for age and sex, with no visible arterial plaque.

### DNA Sequencing and Genetic Analysis

Genomic DNA was isolated from peripheral blood cells using the Gentra Puregene Blood kit (Qiagen, Venlo, The Netherlands). The proband’s DNA sample underwent targeted next-generation sequencing (LipidSeq on the Illumina MiSeq platform), as previously described.^8-10^ The genes for monogenic dyslipidemias on this targeted panel include the following: *LDLR, APOB, PCSK9, LDLRAP1, ABCG5, ABCG8, APOE, LIPA, ABCA1, APOA1, LCAT, CETP, SCARB1, LIPC, LPL, APOC2, APOA5, GPIHBP1, LMF1, APOC3, MTTP*, and *SAR1B.* Variant annotation and VarSeq software (Golden Helix, Bozeman, MT) were used to prioritize rare variants of nonsynonymous sequence ontology, likely to be contributing to disease presentation. Predictive algorithms for pathogenicity, such as Combined Annotation Dependent Depletion (CADD; http://cadd.gs.washington.edu/); Sorting Intolerant From Tolerant (SIFT; http://sift.jcvi.org/); and Polymorphism Phenotyping tool version 2 (PolyPhen-2; http://genetics.bwh.harvard.edu/pph2/) were used to prioritize likely pathogenic variants. Minor allele frequency of each variant was determined based on those cited for Caucasian subpopulations in the Exome Aggregation Consortium database (ExAC; http://exac.broadinstitute.org/); 1000 Genomes database (1000G; http://internationalgenome.org/); and the National Heart, Lung, and Blood Institute Exome Sequencing Project (ESP; http://evs.gs.washington.edu/EVS/). All variants of likely clinical significance were confirmed using Sanger sequencing. The polygenic risk scores (PRSs) for LDL and HDL cholesterol and TG were calculated as described.^11-13^ Briefly, we created a PRS consisting of 10, 14, and 16 single nucleotide polymorphisms associated with LDL cholesterol, HDL cholesterol, and TG levels, respectively, as reported.^11-13^ We calculated a PRS for each lipid variable; for instance, for the TG PRS, the number of TG-raising alleles at a locus (either 0, 1, or 2) was tallied (maximum score 28) and its percentile value relative to the distribution of PRSs in the general population was determined.^11-13^

## Results

Targeted next-generation sequencing for dyslipidemia genes revealed (1) *APOE* E2/E2 homozygous genotype classically described with FDBL, that is, both alleles had p.R130C and p.R176C; (2) in addition, one of the *APOE* E2 alleles contained the rare heterozygous missense variant p.G145D (also known as p.G127D, E1, and apo E-Bethesda), which further alters the net isoform charge by one unit and has been reported as being associated with dyslipidemia^14-17^; (3) a rare unreported heterozygous *APOC2* nonsense variant p.Q92X; and (4) a high PRS for TG levels (16 out of 28 TG-raising alleles) at the 82nd percentile for age and sex. Furthermore, the patient had neither heterozygous pathogenic mutations in genes causing familial hypercholesterolemia (formerly type 2A HLP), nor bi-allelic pathogenic mutations in genes causing recessive dyslipidemias such as familial chylomicronemia syndrome (formerly type 1 HLP), sitosterolemia, or HDL deficiency syndromes such as Tangier or lecithin cholesteryl ester transferase deficiency. There were no DNA sequence abnormalities in *APOC3.*

## Discussion

We report a man with long-standing severe refractory combined dyslipidemia, who presented initially with tuberous xanthomas and equimolar elevations in TC and TG consistent with FDBL. His clinical course was remarkable for a dyslipidemia that has remained refractory to treatment. Recently, the TG elevation has become more prominent. With next-generation sequencing, we uncovered multiple rare genetic “hits” on top of the classical *APOE* E2/E2 genotype, with superimposition of the rare *APOE* missense variant p.G145D on one E2 allele, the rare *APOC2* nonsense variant p.Q92X, and a high PRS for TG (82nd percentile for age and sex).

The concurrent presence of the *APOE* p.G145D mutation may have contributed to the severe dyslipidemia seen here.^12^ The apo E isoform containing this amino acid change in the pre-genomic era was referred to as “E1” and subsequently as “apo E-Bethesda.”^14-17^ Protein sequencing and DNA sequencing revealed that this variant had aspartic acid substituted for glycine at residue 127.^16,17^ This was later renumbered as residue 145 to account for the apo E pro-peptide sequence. The net loss of a positively charged amino acid residue on a background E2 allele encoded by p.G145D results in an apo E isoform that migrates in the E1 position on isoelectrophoretic gels. Other patients with p.G145D came had either European or Turkish ancestry.^14-17^ Defective binding of the p.G145D gene product to cell surface receptors was attributed to the intrinsic binding defect of the E2 allele, which is the background sequence on which the p.G145D mutation resides.^14-17^ Other rare *APOE* variants show a direct causal relationship with dyslipidemia^3^; for instance, *APOE* p.Leu167del is associated with autosomal dominant hypercholesterolemia.^18^ Study of multiple FDBL families with *APOE* p.G145D indicates that this allele requires the presence of additional factors for dyslipidemia to be expressed, as is the case with the typical E2/E2 predisposing genotype.

One such additional factor in the patient reported here could be the *APOC2* p.Q92X missense variant, which prematurely truncates mature apo C-II by ~10%. Apo C-II physiologically activates lipoprotein lipase (LPL), and its activity is counterbalanced by apo C-III, which inhibits LPL.^19^ Other rare nonsense variants affecting apo C-II carboxy terminal peptide sequence are impaired in their ability to activate LPL,^20^ suggesting that the p.Q92X variant would be similarly impaired. Furthermore, our bioinformatic pipeline predicted that this variant was pathogenic.^8-10^ We have previously shown that such heterozygous variants are 4-fold more frequent in cohorts with severe hypertriglyceridemia compared with normolipidemic people.^12^ Heterozygosity for rare loss-of-function variants in genes encoding products involved in lipolysis alone is insufficient to cause severe hypertriglyceridemia, but does predispose to this phenotype in combination with other factors.

The same may apply to the accumulated common variants in genes affecting both TG production and catabolism, as shown by the high PRS for TG in this patient. Patients with severe hypertriglyceridemia are about 3 times more likely to have a high PRS for TG compared with normolipidemic people.^12^ A very high PRS can mimic the effect of a major monogenic mutation.^21^ Again, a high PRS alone is generally not causative for severe hypertriglyceridemia. However, it contributes to susceptibility, which then becomes fully expressed when secondary genetic and non-genetic factors are present.^22,23^ Non-genetic factors in our patient include his increased body mass, mild renal impairment, and proteinuria. The prominence of the TG component of our patient’s combined dyslipidemia may thus be related to the presence of both rare large effect and common small effect variants compounded by non-genetic metabolic contributors.

A recently reported patient with severe FDBL was responsive to treatment with ezetimibe and a PCSK9 inhibitor.^24^ That patient had in common with the current patient both *APOE* E2/E2 homozygosity and a high PRS for TG. In contrast, the patient reported here had additional rare variants—*APOE* p.G145D and *APOC2* p.Q92X—plus increased body mass and renal abnormalities, which cumulatively might have contributed to worsened phenotype. While existing therapies had minimal impact on our patient’s dyslipidemia, it is possible that newer agents that target TG metabolism, such as anti-apo C-III or anti-ANGPTL3 strategies, may be more successful.^25,26^

As next-generation sequencing becomes more routinely used, identification of genetic variants in dyslipidemic patients will increase. In our clinic, all patients consent to targeted gene sequencing and PRS evaluation.^9^ We commonly observe patients with an accumulation of several genetic variants acting collectively and together with non-genetic factors are associated with dyslipidemia.^27^ Attributing pathogenicity to particular variants is not trivial.^28^ An experienced curator is required; we feel that we are conservative when interpreting complex genetic contributors to dyslipidemia.

A potential hazard of the sheer volume of variant data uncovered by this technology is misinterpretation of findings.^28^ For instance, in our patient, no individual genetic variant is directly causative for the dyslipidemia. Each variant contributes to a state of susceptibility, but the risk of disease is probabilistic rather than deterministic. The inheritance of the dyslipidemia phenotype does not follow Mendelian rules, and implications for family members are not straightforward. Because complex polygenic variants cluster in nuclear families, closely related family members are still at risk of dyslipidemia and should be screened biochemically. However, genetic studies will not necessarily be informative. We believe that our panel design and bioinformatic pipeline together with our long experience interpreting genetic data reduces the risk of misinterpretation. This is a general challenge for most complex disorders of adulthood for which next-generation sequencing is now being applied clinically.^28^ Even an apparently straightforward “monogenic” condition like familial hypercholesterolemia has been revealed by modern sequencing to be markedly more complex genetically than was previously believed.^29^

In summary, we present an atypical FDBL patient who is highly refractory to standard medical therapy. Next-generation sequencing uncovered the concurrent presence of multiple genetic variants—both rare and common—that together likely contributed to the patient’s dyslipidemia in combination with non-genetic factors such as obesity, poor diet, and mild renal impairment. His dyslipidemia might be more amenable to treatment with novel biologic agents that target TG-related pathways. Our interpretation of the genetic basis of this patient’s dyslipidemia has been cautious. Furthermore, we recognize the potential that next-generation sequencing can reveal large numbers of variants per genome that play neither a direct nor indirect role in a complex dyslipidemia phenotype, but which nonetheless may still be misinterpreted as being causal.
